# Expression of osteoprotegerin, receptor activator of nuclear factor kappa-B ligand, tumor necrosis factor-related apoptosis-inducing ligand, stromal cell-derived factor-1 and their receptors in epithelial metastatic breast cancer cell lines

**DOI:** 10.1186/1475-2867-12-29

**Published:** 2012-06-18

**Authors:** Vivian Labovsky, Valeria B Fernández Vallone, Leandro M Martinez, Julian Otaegui, Norma A Chasseing

**Affiliations:** 1 Laboratorio de Inmuno-Hematología, Instituto de Biología y Medicina Experimental (IBYME), Buenos Aires, Argentina; 2 Consejo Nacional de Investigaciones, Científicas y Técnicas (CONICET), Buenos Aires, Argentina; 3 Agencia Nacional de Promoción Científica y Tecnológica, Buenos Aires, Argentina; 4 Present address: Vuelta de Obligado 2490, CP 1428, Buenos Aires, Argentina

**Keywords:** OPG, RANKL, TRAIL, SDF-1, Breast cancer cells

## Abstract

**Background:**

While breast cancer (BC) is the major cause of death among women worldwide, there is no guarantee of better patient survival because many of these patients develop primarily metastases, despite efforts to detect it in its early stages. Bone metastasis is a common complication that occurs in 65-80 % of patients with disseminated disease, but the molecular basis underlying *dormancy,* dissemination and establishment of metastasis is not understood. Our objective has been to evaluate simultaneously osteoprotegerin (OPG), receptor activator of nuclear factor kappa B ligand (RANKL), tumor necrosis factor-related apoptosis-inducing ligand (TRAIL), stromal cell-derived factor-1 (SDF-1), and their receptors (R) in 2 human BC cell lines, MDA-MB-231 and MCF-7.

**Methods:**

OPG, RANKL, TRAIL and SDF-1 expression and release, in addition to the expression of their receptors has been investigated using immunofluorescence, immunocytochemistry and ELISA analyses.

**Results:**

MCF-7 cells released higher levels of OPG in conditioned media (CM) than MDA-MB-231 cells; 100 % of both types of cell expressed OPG, RANKL, TRAIL and SDF-1. Moreover, 100 % in both lines expressed membrane RANKL and RANK, whereas only 50 % expressed CXCR4. Furthermore, 100 % expressed TRAIL-R1 and R4, 30-50 % TRAIL-R2, and 40-55 % TRAIL-R3.

**Conclusions:**

MCF-7 and MDA-MB-231 cells not only released OPG, but expressed RANKL, TRAIL and SDF-1. The majority of the cells also expressed RANK, CXCR4 and TRAIL-R. Since these ligands and their receptors are implicated in the regulation of proliferation, survival, migration and future bone metastasis during breast tumor progression, assessment of these molecules in tumor biopsies of BC patients could be useful in identifying patients with more aggressive tumors that are also at risk of bone metastasis, which may thus improve the available options for therapeutic intervention.

## Background

Breast cancer (BC) remains the most common cancer among women in both the United States and worldwide [1-4]. Many individuals with BC develop metastases to secondary organs, e.g. bone marrow (BM) and bone [1-3,5].

The development and spread of tumors are due to the ability of malignant cells to avoid detection and removal by the immune system, grow in non-native sites and avoid programmed or induced cell death [6]. Indeed, 70 % of patients with advanced BC develop metastases in BM and bone (osteolytic lesions) within one year after diagnosis of the primary tumor [2,7]. However, the factors favoring BC growth in both the primary tumor and metastatic sites are unclear, making the mechanisms involved by which primary tumor cells metastasize to secondary organs a major clinical challenge.

Focusing on bone metastasis, there is a vicious cycle between bone resorption and tumor proliferation in which osteoprotegerin (OPG), receptor (R) activator of nuclear factor kappa B (RANK), RANK ligand (RANKL) and tumor necrosis factor (TNF)-related apoptosis-inducing ligand (TRAIL) play a pivotal role [8-10].

OPG, a member of the TNF-R superfamily, binds RANKL, blocking its interaction with RANK and thereby preventing osteoclast differentiation, activation and survival [7,11].

The BC cell line MDA-MB-231 produces sufficient OPG to bind TRAIL, which upregulates RANKL expression [6]. Briefly, OPG secreted by this BC cell line, acting as a paracrine factor, could affect RANKL production, enhancing osteolysis and the perpetuation of this vicious cycle [6]. In human malignancies that metastasize to bone, dysregulation of the RANK/RANKL/OPG pathway can increase the RANKL: OPG ratio, which would favor excessive osteolysis [12-15]. In a mouse model of bone metastasis, the RANKL protein levels in MDA-MB-231 tumor-bearing bones were significantly higher than in tumor-free bones [16]. The resulting tumor-induced osteoclastogenesis and osteolysis were inhibited by recombinant OPG in a dose-dependent manner. Inhibition of RANKL blocked tumor-induced osteolysis and skeletal tumor progression and improved survival in murine models of BC bone metastasis [17]. RANK allows cells to proliferate, migrate and invade other tissues, specifically BM and bone. Additionally, RANK is expressed in many different epithelial tissues and epithelial tumor cells, and *in vitro* stimulation of human BC cell lines (MDA-MB-231, MCF-7 and Hs578T) with RANKL results in concentration-dependent cell migration, which is blocked by recombinant OPG [7]. Moreover, OPG also binds to TRAIL and inhibits its pro-apoptotic effect [18].

TRAIL induces apoptosis through the death receptors DR4/R1 and DR5/R2 that are expressed on the surface of target cells [19-22]. In preclinical models, TRAIL has anticancer activity [23]. Unfortunately, > 50 % of the tumor cells are resistant to TRAIL. In some cases, TRAIL resistance is caused by a high and simultaneous expression of other TRAIL-R-like decoy R (DcR1/R3 and DcR2/R4) and soluble OPG [24]. However, the presence of decoy R cannot explain the lack of response of many cancer cells to antibodies specifically targeting DR4, DR5 or recombinant TRAIL. TRAIL resistance in BC cells has been associated with constitutive endocytosis of death receptors 4 and 5 (R1 and R2) [24]. Thus, it is important to develop new strategies to overcome this type of resistance in tumor cells. Interestingly, some groups have described the ability of subtoxic concentrations of chemotherapeutic drugs to sensitize tumor cells resistant to TRAIL [23,25,26]. Also the anticancer efficacy of TRAIL against BC cells is known to be retained in the bone microenvironment, even in the present of biologically active OPG at a supraphysiologic concentration [18].

Finally, SDF-1, a member of the CXC subfamily of chemokines that mediates several cellular functions (adhesion, survival, proliferation and migration) via interaction with CXCR4, is found at high levels in organs to which BC frequently metastasizes, which include lymph nodes, lungs, liver and bone [27]. CXCR4 is expressed by fibroblasts, endothelial, hematopoietic cells and stromal cells, in different types of cancer cells, such as BC cells, and in numerous types of embryonic and adult stem cells (SCs), which can be chemoattracted by its ligand, SDF-1 [28-31]. CXCR4 expression in tumor cells of several types of carcinomas is correlated with a poor prognosis, e.g. breast and prostate tumors [27,28,32-34]. Furthermore, CXCR4 overexpression in BC cells is correlated with a worse prognosis and decreased patient survival, irrespective of the status of the estrogen-receptor (ER) [35]. Knockdown of CXCR4 expression using small interfering RNA in BC cells decreases *in vitro* cell survival, invasion and proliferation and abrogates *in vivo* tumor growth [28,34,36,37]. In addition SDF-1/CXCR4 in malignant tumors could provide paracrine signals that promote malignant progression, i.e. invasion and cell proliferation that leads to metastasis [28,38]. In contrast, a high level of SDF-1 expression (in a cytoplasmic-dominant pattern) in BC cells seems to be a significant indicator of a better clinicopathological outcome, particularly in patients with ER-positive, HER-2-negative, and lower grade tumors [29]. Moreover, Corcoran et al. [39] detected membrane-bound SDF-1 in MCF-7 and T47D BC cell lines, but not in the MDA-MB-231 cell line, which could be relevant in the interaction with CXCR4-expressing mesenchymal stem cells [39,40]. They also suggested that SDF-1 might occupy CXCR4 in BC cells through autocrine binding, possibly resulting in these cells losing their efficiency in metastasizing to organs with a high concentration of SDF-1. They proposed that CXCR4 antagonists might be ideal for patients who are diagnosed early without lymph node involvement [41]. This type of therapy could be prudent in preventing low numbers of BC cells from entering BM and bone.

A better understanding of the interactions between complex tumor cells and host cells focusing on the bone microenvironment, along with a better understanding of the autocrine and paracrine effects of the factors secreted from tumor cells and bone matrix may facilitate the development of effective strategies that inhibit metastatic disease progression. It is also important to study the early events involved in BC cell entry into the BM.

Therefore, since these ligands and their receptors are molecules involved in the proliferation, survival, migration and possible future BM/bone metastasis of BC cells, studying their simultaneous *in vitro* expression in human BC cell lines, such as the ER-negative MDA-MB-231 (highly invasive and metastatic cells) and ER-positive MCF-7 (weakly invasive and metastatic cells), offers an approach to a better understanding of their involvement in the evolution of BC. Much of the available data about the expression of these factors and receptors in BC cells is to some degree contradictory. Hence, it is important to study the expression and release of these ligands and the expression of their receptors with time (after 1-4 days) and under conditions of arrest and post-stimulation with 10 % fetal bovine serum (FBS) for 48 h.

## Results

### Analysis of the levels of OPG, RANKL, TRAIL and SDF-1 in conditioned media from MDA-MB-231 and MCF-7 cells by ELISA

Since increasing the cell concentration of either BC lines (18x10^3^ or 50x10^3^ cells/cm^2^) did not change the levels of OPG, RANKL, TRAIL and SDF-1 under traditional culture conditions (data not shown), our results represent only the levels using 18x10^3^ cell/cm^2^ in both culture conditions.

**Figure 1:**
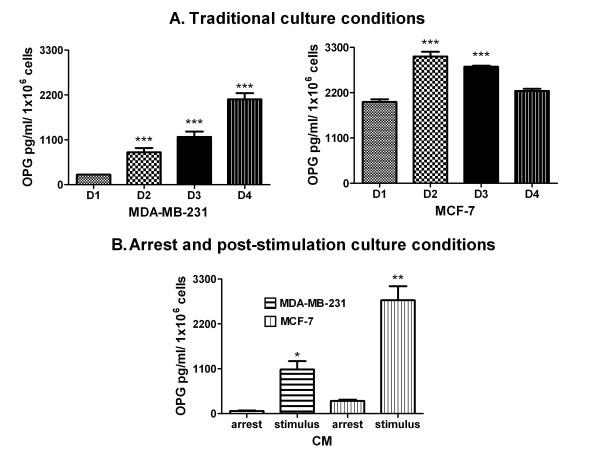
**Release of OPG from BC cell lines under two different culture conditions detected by ELISA.** (**A**) **Traditional culture conditions.** Conditioned media (CM) from the cell cultures at 24, 48, 72 and 96 h [Days (D): D1-D4] from both BC cell lines were used to evaluate the levels of OPG by ELISA. The values of OPG are expressed as $\bar{X}$ pg/ml/ 1x10^6^ cells + standard error (SE). **Statistical significance:** One-way analysis of variance (ANOVA) followed by a Bonferroni post hoc test. Asterisks (*) indicate a significant difference (p <0.05) compared to D1 for each cell line. (**B**) **Arrest and post-stimulation culture conditions.** CM obtained under arrest at 48 h and subsequent stimulation with 10 % SBF for 48 h from both BC cell lines were used to evaluate the levels of OPG by ELISA. The OPG values are expressed as $\bar{X}$ pg/ml/ 1x10^6^ cells + standard error (SE). **Statistical significance:** Unpaired t-test with Welch's correction. Asterisks (*) indicate a significant difference (p = 0.0158, * and p = 0.0056, **) between OPG production during arrest and after stimulation in the MDA-MB-231 and MCF-7 cell lines.

The level of OPG in CM of the MDA-MB-231 cells increased progressively over time, with a maximum at day 4 (D4), whereas in the CM of the MCF-7 cells, the highest level happened after 48 h (day 2, D2) (Figure 1A). MCF-7 cell line released higher levels of OPG than the MDA-MB-231 line at D2 (p < 0.05, Bonferroni post hoc test).

Both cell lines expressed different levels of OPG under conditions of arrest and post-stimulation (Figure 1B). However, the levels of OPG produced after stimulation were higher in the MCF-7 cells compared to the MDA-MB-231 cells (p = 0.0130, unpaired t-test with Welch's correction).

Regardless of the culture conditions and the type of BC cell line used, the levels of RANKL, TRAIL and SDF-1 were below the ELISA detection limit (< 31.25 pg/ml).

### Analysis of the expression of OPG, RANKL and TRAIL in MDA-MB-231 and MCF-7 cells by immunofluorescence

**Figure 2:**
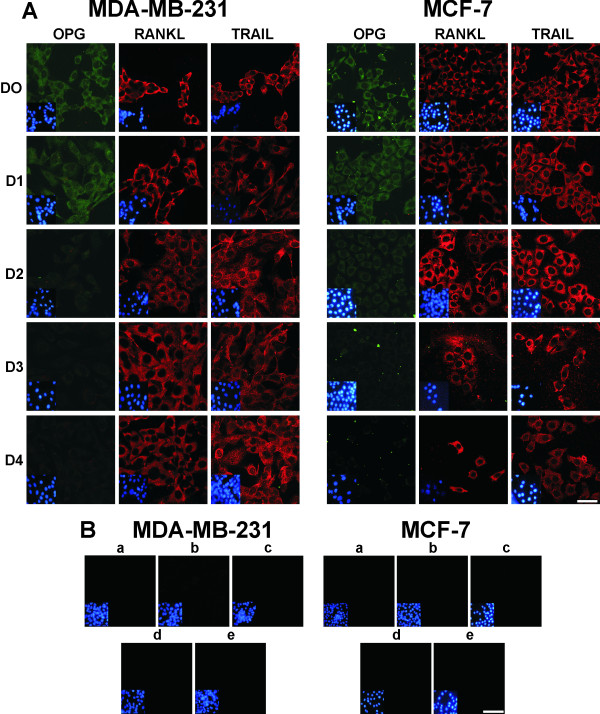
**Immunofluorescence detection of OPG, RANKL and TRAIL in both BC cell lines grown under Traditional culture conditions. (A)** Immunofluorescence staining for OPG, RANKL and TRAIL was positive in both BC cell lines. D: day; D0: cells at time zero; D1, D2, D3 and D4: cell lines grown for 24, 48, 72 and 96 h, respectively. The insert shows nuclear DNA stained with DAPI. The scale bar represents 30 μm. **(B)** Both BC cell lines were grown until D4 (96 h) and incubated either without a primary Ab and with the followings Abs: **a)** Cy3-labeled goat anti-mouse IgG, **b)** FITC-labeled goat anti-rabbit IgG, **c)** an irrelevant Ab as a negative isotype control, such as IgG1, **d)** mouse Igs and **e)** rabbit Igs. In controls **c** and **d**, the cell lines were stained with a Cy3-labeled goat anti-mouse IgG Ab, and in control **e**, they were stained using a FITC-labeled goat anti-rabbit IgG Ab. The insert shows nuclear DNA stained with DAPI. The scale bar represents 30 μm.

*In vitro* traditional culture condition results in both BC cell lines expressing similar levels of OPG, RANKL and TRAIL (Figure 2A). MDA-MB-231 and MCF-7 cells displayed a decrease in OPG expression at day 2. OPG, RANKL and TRAIL were expressed in 100 % of the BC cells. No immunostaining was observed when the MDA-MB-231 and MCF-7 cells were incubated either without a primary antibody (Ab) or with an irrelevant Ab as a negative isotype control. Figure 2B shows only the results obtained from the two BC cell lines grown until D4 as representative results.

**Figure 3:**
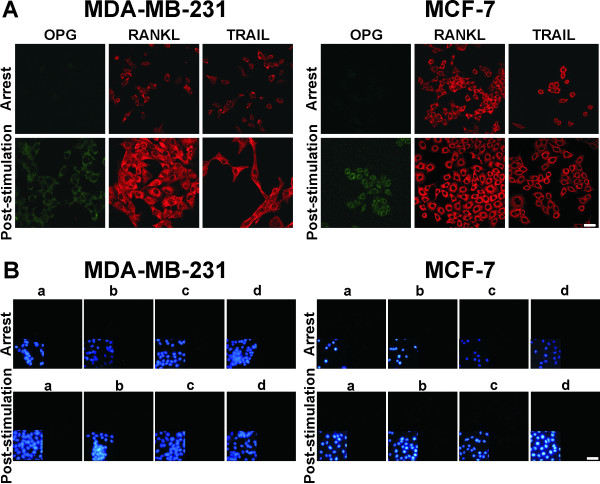
**Expression of OPG, RANKL and TRAIL in both BC cell lines.** (**A**) Immunofluorescence staining for OPG, RANKL and TRAIL in both BC cell lines grown under arrest and post-stimulation culture conditions. The scale bar represents 50 μm. (**B**) The MDA-MB-231 and MCF-7 cell lines were grown under arrest and post-stimulation culture conditions and incubated either without a primary Ab and with the followings Abs: **a**) Cy3-labeled goat anti-mouse IgG, **b**) FITC-labeled goat anti-rabbit IgG, **c**) an irrelevant Ab as a negative isotype control, such as IgG1, and **d**) rabbit Igs. In control **c,** the cell lines were stained with a Cy3-labeled goat anti-mouse IgG Ab, and in control **d,** they were stained with a FITC-labeled goat anti-rabbit IgG Ab. The insert shows nuclear DNA stained with DAPI. The scale bar represents 30 μm.

RANKL and TRAIL were also expressed in 100 % of the MDA-MB-231 and MCF-7 cells, under conditions of both arrest and post-stimulation (Figure 3A). Independently of the cell type used, this expression/cell was higher post-stimulation. Regarding the production of OPG its expression/cell was minimal under arrest conditions but increased post stimulation. No immunofluorescence was observed when the primary Ab was omitted or other negative controls were used (Figure 3B).

### Evaluation of the expression of membrane RANKL in MDA-MB-231 and MCF-7 cells by immunocytochemistry

**Figure 4:**
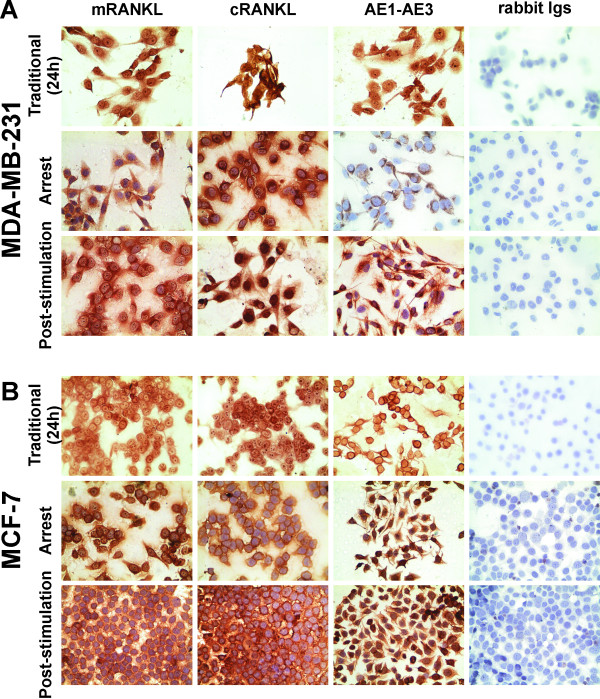
**Expression of membrane RANKL on BC cell lines under the two different culture conditions.** (**A**) Immunocytochemistry staining for mRANKL in the MDA-MB-231 cell line under both culture conditions (x 400 magnification). (**B**) Immunocytochemistry staining for mRANKL in the MCF-7 cell line under both culture conditions (x 400). Positive controls were performed in both BC cell lines incubated with anti-RANKL (cytoplasmatic RANKL = cRANKL, evaluated previously) and anti-AE1AE3 (a pan-cytokeratin epithelial marker). No staining was observed in both BC cell lines incubated with an irrelevant rabbit Ig Ab as a negative isotype control. Nuclei were counterstained with hematoxylin (purple).

Independently of the culture conditions or the cell concentration plated, RANKL was undetectable in the CM of the MDA-MB-231 and MCF-7 cells. Membrane expression of RANKL (mRANKL) should be evaluated as its functions could be assessed not only as soluble factor but also as a membrane protein via cell-to-cell contact through RANK. Immunocytochemistry analysis of the MDA-MB-231 and MCF-7 cells showed positive and similar expression of membrane and cytoplasmatic RANKL (cRANKL) in 100 % of cells under both culture conditions. Figure 4A and B show the results from both BC cell lines grown under traditional culture conditions for 24 h (day 1, D1) and arrest and post-stimulation culture conditions. Positive expression for AE1AE3 was used as a positive control. No immunostaining was observed in any of the cells when they were incubated without specific primary Abs or with other negative control.

### Evaluation of the expression of RANK, SDF-1 and CXCR4 in MDA-MB-231 and MCF-7 cells by immunocytochemistry

**Figure 5:**
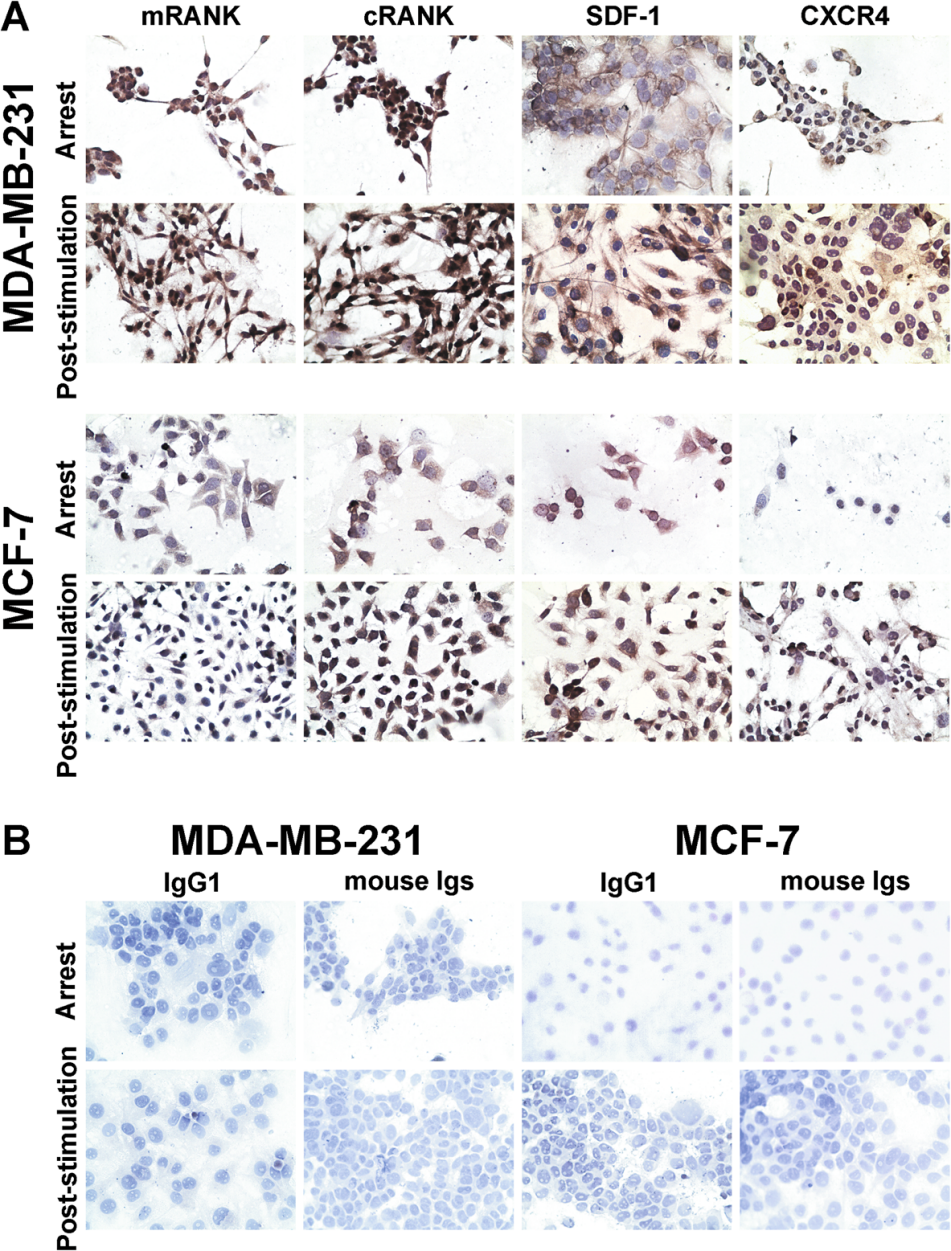
**Expression of membrane and cytoplasmic RANK (mRANK/cRANK), SDF-1 and CXCR4 in both BC cell lines.** (**A**) Immunocytochemistry staining for RANK, SDF-1 and CXCR4 in MDA-MB-231 and MCF-7 cells grown under arrest and post-stimulation culture conditions (x 400 magnification). (**B**) The MDA-MB-231 and MCF-7 cell lines were incubated with irrelevant IgG1 and mouse Igs Abs as negative isotype controls (x 400 magnification). Nuclei were counterstained with hematoxylin (purple).

Immunocytochemistry analysis of membrane and cytoplasmic RANK (mRANK and cRANK) displayed 100 % positive expression in MDA-MB-231 and MCF-7 cells under both culture conditions. Figure 5A and B show the results from the two BC cell lines grown under arrest and post-stimulation culture conditions.

One hundred of BC cells expressed SDF-1 under both culture conditions. In contrast, 50 % of these cells showed a weak CXCR4 expression under both culture conditions. But, there was an increase of expression after stimulation and on days 1 to 4 under traditional culture conditions. Figure 5A and B show the results obtained for BC cells grown under arrest and post-stimulation culture conditions. No staining was observed in the negative controls (Figure 5B).

### Evaluation of the expression of TRAIL and its receptor in MDA-MB-231 and MCF-7 cells by immunocytochemistry

**Figure 6:**
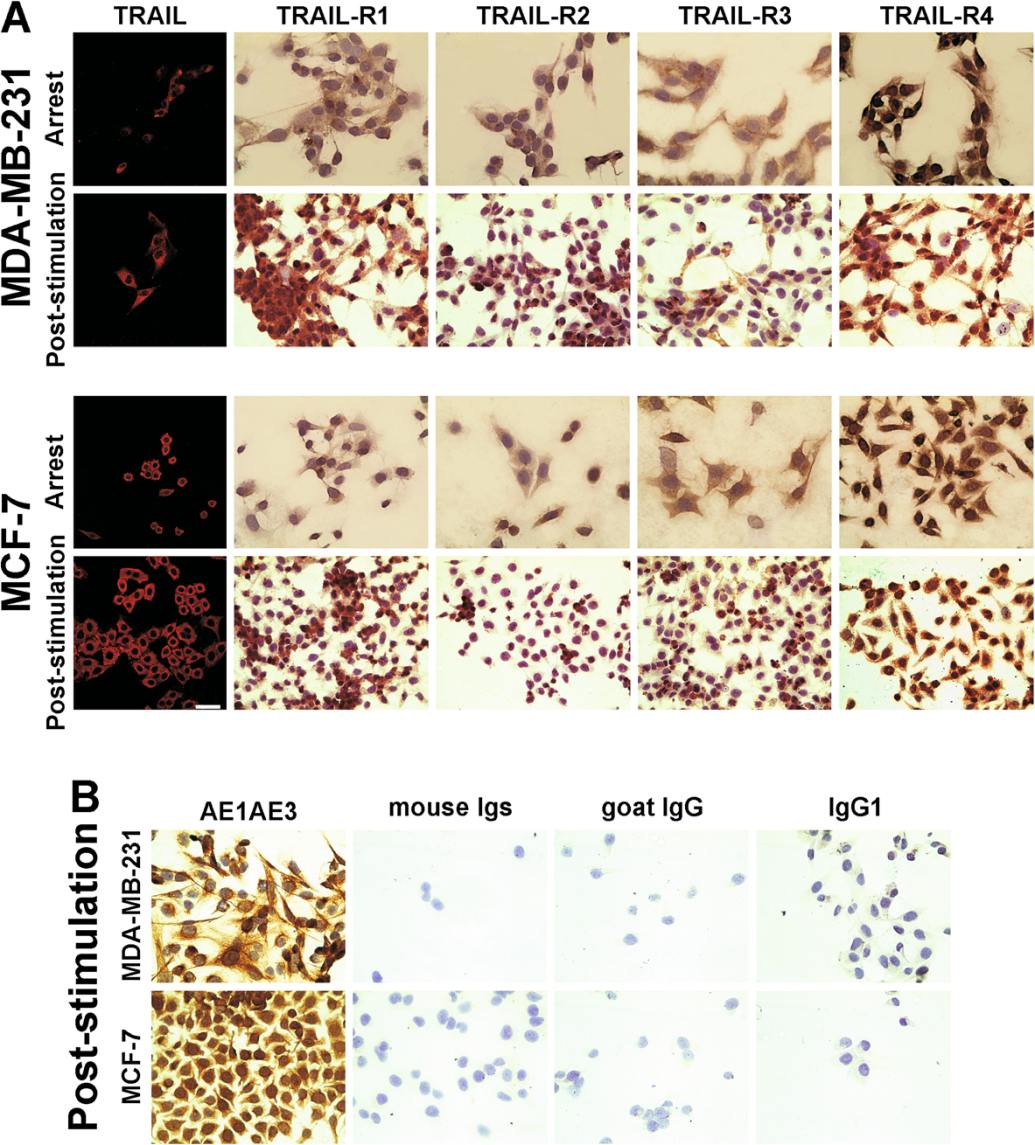
**Expression of TRAIL, TRAIL-R1, TRAIL-R2, TRAIL-R3 and TRAIL-R4 in both BC cell lines.** (**A**) Immunocytochemistry staining for TRAIL, TRAIL-R1, TRAIL-R2, TRAIL-R3 and TRAIL-R4 in the MDA-MB-231 and MCF-7 cell lines grown under arrest and post-stimulation culture conditions (x 400 magnification). (**B**) The MDA-MB-231 and MCF-7 cell lines were incubated with anti-AE1AE3 (positive control) and with irrelevant mouse Igs, goat IgG and IgG1 Abs as negative isotype controls (x 400 magnification). Nuclei were counterstained with hematoxylin (purple).

Figures 6A and B show the results obtained from the two BC cell lines grown under arrest and post-stimulation culture conditions. Immunocytochemistry staining of MDA-MB-231 and MCF-7 cells exhibited positive and similar expression patterns of TRAIL-R1 and R4 in 100 % of cells in cultures of both lines (Figure 6A). However, TRAIL-R2 and TRAIL-R3 were weakly expressed. TRAIL-R2 expression was stronger in MDA-MB-231 cells than in MCF-7 cells, with 30 to 50 % of cells showing staining in cultures of both cell lines. TRAIL-R3 was expressed with the same intensity in culture, showing 40 to 55 % positive cells.

Positive expression of AE1AE3 (used as a positive control) was observed. In cultures where the primary Ab was omitted or other negative controls were used, negligible staining was observed (Figure 6B).

## Discussion

Bone metastasis is a common event in advanced breast cancer. Once tumors metastasize to bone, they are usually incurable, with a five-year survival rate of 20 % [42].

MCF-7 and MDA-MB-231 cell lines, extracted from pleura, are metastatic and belonged to BC patients at an advanced clinical stage. In addition, Corcoran et al. found a low invasivity preference of MCF-7 and T47D cells to BM, but MDA-MB-231 cells are highly metastatic to lung and BM [39]. Based on these background findings, the results presented in this work may contribute to *in vitro* analysis of the expression and release of factors involved in the BM/bone metastasis of BC cells, such as OPG, RANKL, TRAIL, SDF-1 and their receptors.

OPG and its two ligands, TRAIL and RANKL, are expressed at different levels in normal breast tissue and breast tumor tissue [43]. However, it is not fully understood their role during the development of human BC, and which types of cells produce them within the tumor microenvironment. Therefore, OPG could exert either anti- or pro-tumoral effects in cells based on whether it binds RANKL or TRAIL, which depends on the relative concentrations of the two cytokines in BC tissue during tumor progression [8,11,44,45]. OPG binding to TRAIL inhibits the apoptosis of some BC cells, increasing their survival. Nevertheless, OPG binding to RANKL inhibits osteoclastogenesis and in some BC cells, decreases the ability to migrate, especially to BM/bone, due to inhibiting the interaction between RANKL and RANK [7,46]. RANKL is abundantly expressed in BM/bone [7]. Recently, Santini et al. demonstrated that RANK expression in primary tumors from BC patients is a predictive marker of both the occurrence of BM/bone metastasis and of shorter skeletal disease-free survival [47].

TRAIL acts as an apoptotic factor in osteoclasts and in BC cells (ER negative) [48,49]. In primary breast tumor and metastatic sites, TRAIL is released by BC cells, activated fibroblasts, macrophages, lymphocytes and post- TNF-α/interferon-α/-γ stimulated mesenchymal stem cells (MSCs) [8,43,45,50].

The function of OPG released by a tumor after migration to BM/bone is not fully understood because of the low levels of secretion compared to those produced by MSCs from BM and osteoblasts [44]. Accordingly, we found that both the MDA-MB-231 and MCF-7 cell lines, independent from being ER positive or negative, expressed and released OPG. The OPG levels detected in the CM from both culture conditions were lower than the levels found in previous studies from our laboratory working with the CM of MSCs from the BM of healthy volunteers and untreated advanced BC patients free of BM and bone metastases [51,52]. Therefore, we can conclude that when BC cells first enter BM/bone, they release OPG that may be important via binding to TRAIL secreted by osteoclasts or other cells of the stromal microenvironment. Moreover, BC cells can play a role in the distance releasing of OPG or other factors to the circulation, hence exerting its action in BM/bone. Thus, BC cells may inhibit their own and osteoclasts apoptosis and further contribute to the spontaneous osteoclastogenesis observed by our group in the peripheral blood and BM of untreated advanced BC patients prior to bone metastasis [51,52]. However, this last interaction cannot significantly modify the production of osteoclasts because it is triggered by the hematopoietic microenvironment, especially MSCs and osteoblasts, prior to the arrival of tumor cells and of the development of bone metastasis [51,52].

Furthermore, OPG can be regulated by sulfated proteoglycans (PGs), which inhibit the adherence and resorptive activity of osteoclasts [8,53]. The capture of OPG in the extracellular matrix of BM/bone favors bone osteolysis, further contributing to bone disorders leading to the appearance of metastasis. The PG-OPG interaction blocks OPG activity via preventing binding to TRAIL and RANKL as well as decreasing its half-life [8,53,54]. Therefore, the OPG anti- or pro-tumor activity in the BM/bone microenvironment is determined by the relative concentrations of each of its ligands (RANKL, TRAIL and sulfated PG) [8]. In contrast, recent data indicated that the role of OPG released by BC cells injected intratibially into normal mice preserved the integrity of bone and prevented BC-induced bone destruction because its principal role is binding RANKL *in vivo*[18]. Finally, OPG increases endothelial cell survival and induces angiogenesis, favoring further development of primary tumors and the appearance of metastasis, including to BM and bone [11].

As stated, BC cells from the MCF-7 and MDA-MB-231 cell lines released OPG, independent of the culture conditions used. MCF-7 line released was greater and faster as a function of time. These results are consistent with those of other authors who found that the production of OPG is higher in ER-positive cells in primary tumors than in ER-negative cells [43,55]. In addition, Holen et al. have observed that after culturing the MDA-MB-231 line for 72 h, the cells produced 768 ± 64 pg/ml of OPG [55], which is very similar to the levels we obtained for this line on the third day (614.20 ± 161 pg/ml, traditional culture conditions). In contrast, other authors have detected OPG levels of approximately 1,750 pg/ml in MDA-MB-436 cells and comparable levels in MDA-MB-231, but they have not observed OPG in CM from MCF-7 cells [44].

We detected intracytoplasmic expression of OPG, RANKL and TRAIL in both cell lines, observing a decrease in the expression of OPG from the second day (traditional culture conditions) in both cell lines. This correlates with higher levels of OPG in the CM. Kapoor et al. observed that the expression of OPG in MDA-MB-231 cells is directly correlated with the colonization and homing potential related to bone and not with metastasis to other organs [56].

Based on these findings, it appears that OPG has a multifactorial role in the development of osteolytic-type bone metastasis, as in BC. An increase in the RANKL/OPG ratio favors the formation of osteoclasts within the bone microenvironment and is a bad prognostic factor in BC patients [12]. In the present study, we were not able to quantify the level of soluble RANKL in the CM of BC cells. Nevertheless, we found that 100 % of BC cells expressed membrane RANKL under both culture conditions, independent of the tumor cell line used, allowing these cells to proliferate, migrate and specifically invade BM and bone. Brown et al. showed that cells that metastasize to bone exhibit higher RANKL expression than cells that metastasize to other organs [57]. Therefore, BC cells could migrate to BM/bone and promote the differentiation of preosteoclasts into osteoclasts, thus further promoting the osteolytic process that leads to the emergence of bone metastasis. This suggests that the most important event for the bone metastasis establishment is the increase in osteoclast formation and bone resorption processes that finally leads to the invasion and proliferation of BC cells in bone.

We showed that 100 % of the cells from the MDA-MB-231 and MCF-7 cell lines expressed RANK and SDF-1 under both culture conditions, despite showing no detectable levels of this last factor released into the CM. Moreover, analysis of CXCR4 expression exhibited positive weak staining in 50 % of the cells from both BC cell lines under both culture conditions. These results suggest that cells expressing RANK and CXCR4 have the ability to migrate to BM through the action of RANKL and SDF-1 released by BM stromal cells, fibroblasts or osteoblasts [4,58-60].

Neville-Webbe et al. have shown that hormone independent TRAILS’ sensitive BC cell lines cease being sensitive in the presence of recombinant OPG, thereby enhancing tumor cell survival [44]. In contrast, other authors have reported that the anti-cancer efficacy of TRAIL is retained in the presence of high, biologically active concentrations of OPG *in vivo*[18]. These findings showed the importance of measuring the levels of OPG, TRAIL and TRAIL-R in BC patients, particularly those that are ER negative. Further investigation of the regulation of TRAIL by OPG could aid in designing new therapeutic strategies, where the apoptotic action of TRAIL could be increased in BC cells at the early stages of the disease.

Regarding the release of TRAIL, the results of the present study showed levels below the minimum level detectable by ELISA in the CM from both tumor cell lines. It is probable that *in vitro*, some of the multiple factors needed for the release of TRAIL by BC cells are absent, as this situation is very different from that *in vivo*.

Yagita et al. have reported that the absence of total TRAIL-R1 and R2 protein on the cell surface is sufficient to account for the failure of apoptosis [21]. Thus, evaluation of expression of these receptors could serve as a potential predictive marker of TRAIL sensitivity in BC cells. Moreover, positive TRAIL-R4 expression is correlated with tumoral grade in BC patients with invasive ductal carcinoma [61], the expression of TRAIL-R is associated with the resistance of MCF-7 cells to the action of TRAIL and the expression of TRAIL-R3/R4 could compete with TRAIL-R1 and/or -R2 to binding TRAIL blocking apoptosis signaling [62,63]. It appeared to be important to assess TRAIL-R expression in the MDA-MB-231 and MCF-7 cell lines to consolidate the results under the two different culture conditions. Our data showed that 100 % of the cells from both lines expressed TRAIL-R1 and TRAIL-R4. However, we found that only 30 to 55 % of these cells weakly express TRAIL-R2 and TRAIL-R3, being stronger the TRAIL-R2 expression in MDA-MB-231 than in MCF-7 cell lines.

This study demonstrates the importance of obtaining our own expression results of OPG, RANKL, RANK, TRAIL, TRAIL-R (R1, R2, R3 and R4), SDF-1 and CXCR4 in cultures from the human BC cell lines MDA-MB-231 and MCF-7 in different culture conditions. Taking these findings together with those previously obtained, allows us to understand some of the factors and mechanisms triggered prior to the appearance of bone metastasis and during the interactions between tumor cells, MSCs from BM and blood components in greater detail. It will be of interest to study simultaneously these molecules in tumor biopsies as well as in tumor epithelial cells isolated from BC patients. They may be useful in identifying patients with bone risk, and thus, they may improve decision related to the different treatments to combat cancer progression.

Finally, despite the advances in knowledge about these molecules, many questions remain unresolved, particularly regarding the conflicting roles of OPG in bone metastasis and the possible use of OPG as a potential therapeutic factor for tumors that produce metastases in BM and bone. In conclusion, MCF-7 and MDA-MB-231 cell lines not only released OPG but also expressed RANKL, TRAIL and SDF-1. The majority of these cells also expressed RANK, CXCR4 and TRAIL-R. Since these ligands and their receptors are involved in the regulation of breast tumor progression, the simultaneous assessment of these molecules in tumor biopsies of BC patients, particularly in the early clinical pathological stage, could be useful in identifying patients with more aggressive tumors that are also at risk of bone metastasis, which may thus improve the available options for therapeutic intervention.

## Methods

### Breast cancer cell lines

The MDA-MB-231 and MCF-7 lines were maintained in DMEM/F12 with 100 IU/ml penicillin-G, 100 μg/ml streptomycin sulfate, 25 μg/ml amphotericin B, 2 mM L-glutamine (supplemented DMEM/F12) (Gibco/Life Technologies, Grand Island, NY, USA) and 10 % fetal bovine serum (FBS; Natocor, Argentina), complete DMEM/F12, at 37 °C under 5 % CO_2_. In the case of the MCF-7 line, 2 μg/ml of humanized porcine insulin (Laboratories Beta, Argentina) was added. The medium was changed every 3-4 days. The cells were used up to passage 4.

To better compare the expression of OPG, RANKL, TRAIL, SDF-1 and their receptors (RANK, TRAIL-R1-4 and CXCR4), both BC cell lines were grown under two culture conditions: A) traditional and B) adherence, arrest and post-stimulation.

### Traditional culture conditions (A)

The BC cell lines (18x10^3^ or 50x10^3^ cells/cm^2^) were cultured in complete DMEM/F12 for 24, 48, 72 or 96 h. Then, the CM were harvested from BC cell lines on days 1 to 4 (D1, D2, D3 and D4), centrifuged at 300 *g* for 15 min and stored at -20 ° C until use. This experiment was repeated four times.

### Adherence, arrest and post-stimulation culture conditions (B)

The BC cell lines (18x10^3^ cells/cm^2^) were cultured in complete DMEM/F12 without phenol red (D2906, Sigma, St. Louis, MO, USA) for 24 h (adherence) and then with supplemented DMEM/F12 without phenol red, insulin and FBS for an additional 48 h (arrest). Finally, the cells were cultured with complete DMEM/F12 for 48 h (stimulus). The CM from the arrest and post-stimulation culture conditions were harvested, centrifuged at 300 *g* for 15 min and stored at -20 ° C until use. The experiment was repeated four times.

### ELISA assays

The CM from both culture conditions (**A** and **B**) were used to evaluate OPG, RANKL, TRAIL and SDF-1 levels via ELISA methodology. Cells from both culture conditions (**A** and **B**) were harvested using a solution of trypsin-EDTA (0.05-0.02 % in PBS, Gibco) with the purpose of evaluating the number of total cells/culture; the levels of these factors were then expressed per 1x10^6^ cells.

OPG, RANKL and SDF-1 levels were measured in the CM using commercial ELISA kits (DY805, R&D, Minneapolis, MN, USA; RHF740CKC, Antigenix America, Huntington Station, NY, USA; and DY350, R&D, respectively). TRAIL was measured using an ELISA kit developed in our laboratory. This procedure was performed according to the manufacturer’s recommendations for human TRAIL/TNFSF10 (DY375, R&D) but using anti-human TRAIL (MAB3751, R&D) as capture Ab; recombinant human TRAIL (rhTRAIL, 375-TL, R&D, at 31.25 to 2,000 pg/ml) to produce a standard curve; and anti-human TRAIL (BAF375, R&D) as a detection Ab together with streptavidin-peroxidase (S5512, Sigma).

The enzyme activity was revealed using a 3,3′,5,5′-Tetramethylbenzidine (TMB) substrate (Sigma), and it was abolished using 1 N HCl (Merck). The absorbance was read at 450 nm using a microplate reader set (BIO-RAD, Hercules, CA, USA). Supplemented DMEM-F12 with 10 % FBS (D0) was used as a control, and its value was eliminated as a test target. Samples were assayed in triplicate. The CM were assayed from four experiments run under both culture conditions (**A** and **B**). The levels of the OPG, RANKL, TRAIL and SDF-1 proteins from the ELISA analyses were calculated in pg/ml/1x10^6^ cells.

### Immunofluorescence assays

The BC cells from both culture conditions (**A** and **B**) were used to evaluate the expression of OPG, RANKL and TRAIL via immunofluorescence. The experiment was repeated four times for each culture condition.

BC cells (18x10^3^ cells/well/130 μl) were grown on multi-test slides (096041805, MP Biomedicals, Santa Ana, CA, USA) during both culture conditions (**A** and **B**). Then, the BC cells were washed with PBS and fixed with 4 % paraformaldehyde for 30 min. After blocking in PBS-1 % BSA, the cells were incubated with the following primary human Abs: anti-OPG (AB21259, Chemicon, Billerica, MA, USA), anti-RANKL (MAB626, R&D) and anti-TRAIL (MAB687, R&D). The presence of OPG was visualized using FITC-labeled goat anti-rabbit IgG (H + L) (FI-1000, Vector Lab), and the presence of RANKL and TRAIL was visualized using Cy3-labeled goat anti-mouse IgG (H + L) (111-165-146, Jackson). Finally, mounting media with DAPI was used to visualize nuclei (Vectashield solution, H-1500, Vector Lab). Confocal microscopy analyses were performed using a Nikon C1 laser-scanning confocal microscope (Plan Apo 40x/0.95). The acquisition software used was MetaMorph image analysis software.

BC cells were also incubated without primary Abs; with irrelevant IgG1 [mouse IgG1 isotype (X0931, Dako, Carpinteria, CA, USA)]; with normal mouse Igs (08-6599, Zymed, San Francisco, CA, USA); or with normal rabbit Igs (X0936, Dako) as a negative isotype control. Each sample was assayed in quadruplicate.

### Immunocytochemistry

The BC cells from both culture conditions (**A** and **B**) were used to evaluate the expression of RANKL, RANK, TRAIL-R1-4, SDF-1 and CXCR4 via immunocytochemistry assays. The experiment was repeated four times for each culture condition.

BC cells (18x10^3^ cells/well/130 μl) were fixed in 4 % paraformaldehyde, except for cells that were used to evaluate the expression of mRANKL and mRANK. After blocking endogenous peroxidase activity and non-specific sites (PBS-BSA 1 %), the BC cells were incubated with the following primary human Abs: anti-RANKL (AB1862, Chemicon), anti-RANK (MAB683, R&D), anti-TRAIL-R1 (AF347, R&D), anti-TRAIL-R2 (MAB6311, R&D), anti-TRAIL-R3 (MAB6301, R&D), anti-TRAIL-R4 (AF633, R&D), anti-SDF-1 (MAB350, R&D), anti-CXCR4 (MAB172, R&D) and anti-AE1AE3 as a positive control (IR053, Dako). According to the manufacturer’s recommendations, a peroxidase-based immunocytochemistry staining method (K0690, Dako) was used for primary Ab detection, and a 3,3′-diaminobenzidine tetrahydrochloride (DAB) substrate system (K3468, Dako) was used as the chromogen.

Negative controls without primary Abs; with an irrelevant Ab as a negative isotype control [mouse IgG1 isotype (X0931, Dako)]; with normal mouse Igs (08-6599, Zymed); with normal goat IgG (AB-108-C, R&D); or with normal rabbit Igs (X0936, Dako) were used to assess non-specific staining. Each sample was assayed in quadruplicate.

### Statistical analysis

Data obtained from ELISA were expressed as $\bar{X}$ ± standard error (SE) pg/ml/1x10^6^ cells. The statistical significance of the differences in the measured values among groups was evaluated by one-way analysis of variance (ANOVA) followed by a Bonferroni post hoc test. For individual comparisons, an independent unpaired t-test with Welch's correction was used. A difference was considered statistically significant if p < 0.05.

## Abbreviations

Ab: Antibody; AE1AE3: Anti-cytokeratin monoclonal antibodies; ANOVA: Analysis of variance; BC: Breast cancer; BM: Bone marrow; CM: Conditioned media; CXCR4: C-X-C chemokine receptor type 4; C: Cytoplasmatic; Day: D; Dc: Decoy; ER: Estrogen-R; FBS: Fetal bovine serum; HER-2: Epidermal growth factor receptor 2; M: Membrane; M-CSF-R: Macrophage colony stimulating factor receptor; M-CSF: M-CSF-R ligand; MSC: Mesenchymalstem cells; OPG: Osteoprotegerin; PG: Proteoglycans; R: Receptor; RANK: Activator of nuclear factor kappaB; RANKL: RANK ligand; Rh: Recombinant human; SC: Stem cells; SDF-1: Stromal cell-derived factor 1; SE: Standard error; TNF: Tumor necrosis factor; TRAIL: TNF-related apoptosis-inducing ligand.

## Competing interests

The authors declare that they have no competing financial interests.

## Authors’ contributions

VL has contributed to the conception and design of the study, performed the statistical analysis, interpretation of data and drafted the manuscript. VBFV participated in the design of the study, performed the statistical analysis, drafted and revised the article. LMM cultured the cell lines, drafted and revised the article. OJ cultured the cell lines and carried out the western blot assay. ANC participated in the design of the study and in the revision of the article as well as in the final approval of the version to be submitted. All authors read and approved the final version of the manuscript.
